# Effects of self-rated health on sick leave, disability pension, hospital admissions and mortality. A population-based longitudinal study of nearly 15,000 observations among Swedish women and men

**DOI:** 10.1186/1471-2458-12-1103

**Published:** 2012-12-22

**Authors:** Christina Halford, Thorne Wallman, Lennart Welin, Annika Rosengren, Annika Bardel, Saga Johansson, Henry Eriksson, Ed Palmer, Lars Wilhelmsen, Kurt Svärdsudd

**Affiliations:** 1 Department of Public Health and Caring Sciences, Family Medicine and Preventive Medicine, Uppsala University, Uppsala, Sweden; 2 R&D Centre/Centre for Clinical Research, Section of Primary Care, Sörmland County Council, Eskilstuna, Sweden; 3 Department of Heart and Lung Diseases, Sahlgren Academy, Gothenburg, Sweden; 4 Department of Epidemiology, AstraZeneca R&D, Mölndal, Sweden; 5 Uppsala University, Department of Economics, Uppsala, Sweden; 6 National Social Insurance Agency, Stockholm, Sweden

## Abstract

**Background:**

Simple global self-ratings of health (SRH) have become increasingly used in national and international public health monitoring, and in recent decades recommended as a standard part of health surveys. Monitoring developments in population health requires identification and use of health measures, valid in relation to targets for population health. The aim of the present study was to investigate associations between SRH and sick leave, disability pension, hospital admissions, and mortality, adjusted for effects of significant covariates, in a large population-based cohort.

**Methods:**

The analyses were based on screening data from eight population-based cohorts in southern and central Sweden, and on official register data regarding sick-leave, disability pension, hospital admissions, and death, with little or no data loss. Sampling was performed 1973–2003. The study population consisted of 11,880 women and men, age 25–99 years, providing 14,470 observations. Information on SRH, socio-demographic data, lifestyle variables and somatic and psychological symptoms were obtained from questionnaires.

**Results:**

There was a significant negative association between SRH and sick leave (Beta −13.2, p<0.0001, and −9.5, p<0.01, in women and men, respectively), disability pension (Hazard ratio 0.77, p<0.0001 and 0.76, p<0.0001, in women and men, respectively), and mortality, adjusted for covariates. SRH was also significantly associated with hospital admissions in men (Hazard ratio 0.87, p<0.0001), but not in women (Hazard ratio 0.96, p0.20). Associations between SRH on the one hand, and sick leave, disability pension, hospital admission, and mortality, on the other, were robust during the follow-up period.

**Conclusions:**

SRH had strong predictive validity in relation to use of social insurance facilities and health care services, and to mortality. Associations were strong and robust during follow-up.

## Background

In recent decades there has been increasing interest in development of health measures for monitoring changes in population health and health care needs. Monitoring developments in population health requires identification and use of health measures valid in relation to targets for population health.

Global self-rated health (SRH), a single-item question with good overall reliability [1], and a powerful predictor of morbidity [2-4] and mortality [5,6], has during the past decades become extensively used as a health measure in national and international public health monitoring and recommended as a standard part of health surveys [7,8]. Associations between SRH and morbidity and mortality have been extensively studied, however, less is known concerning associations between SRH and other relevant health measures.

Inverse associations between SRH and sick leave [9-12], disability pension [13-15], and utilization of health care [16-19] have consistently been reported. However, concerning sick-leave results are mainly based on populations consisting of employees from specific sectors of the work-force, and results based on data from population based studies are needed. Furthermore, concerning hospital admissions, studies are mainly based on self-reported data concerning health care utilization, and there is a need for studies using official register based data.

Another important issue is related to the duration of the predictive power, that is, for how long a single SRH measurement affects outcome. There is a void of studies investigating the stability and duration of the observed association between SRH on the one hand, and sick leave, disability pension or hospital admission on the other. SRH has been associated with mortality in follow-up periods of up to 20 years [20], with stronger associations observed in short-term as compared to longer follow-up periods [21,22]. Finally, similar associations between SRH and mortality have been observed in women and men [21,22], although mixed findings have also been reported [20].

The aim of the present study was to analyze the effects of SRH on sick leave, disability pension, hospital admission, and mortality, in a large population-based cohort of adult women and men, using outcome data based on official registers. Furthermore, the aim was to estimate for how long during follow-up the effect of SRH persists.

## Methods

### Study population

Data from eight ongoing population cohort studies in Sweden, with baseline investigations performed between 1973 and 2003, were used for this study. The study population has previously been described in detail [23,24]. Briefly, random samples based on predefined specifications concerning age, sex, and area of residence, were drawn from the national population register. The Men Born in 1913 subsample consisted of a random third of the male population aged 60 in the city of Gothenburg, Sweden, in 1973, and the Men Born in 1923 subsample consisted of a random tenth of the male population aged 50 in Gothenburg Table 1. Survivors in the samples were invited to re-examinations in 1980, 1988 and 1993.

**Table 1:** Cohorts

**Sub**-**populations**	**Investigation**		**Sex**	**Age range**	**Sample size**	**Responders**	**Response rate**	**Investigation procedure**
	**Year**	**Place**			**n**	**n**	**%**	
Men born in 1913	1973	Gothenburg	men	60	1,009	830	82.3	Q + ME
	1980	Gothenburg	men	67	923	707	76.6	Q + ME
	1988	Gothenburg	men	75	702	463	66.0	Q + ME
	1993	Gothenburg	men	80	447	272	60.9	Q + ME
Men born in 1923	1973	Gothenburg	men	50	292	226	77.4	Q + ME
	1980	Gothenburg	men	57	278	188	67.6	Q + ME
	1988	Gothenburg	men	65	265	162	61.1	Q + ME
	1993	Gothenburg	men	70	226	143	63.3	Q + ME
Eskil	1986	Eskilstuna	men	30-54	625	459	73.4	PQ
Public Health Cohort	1993	Uppsala	women	25-99	2,999	2,249	75.0	PQ
			men	25-94	3,001	2,156	71.8	
Beda II	1997	Gothenburg	women	56-82	994	908	91.3	Q + ME
Uppsala-Örebro Women Study	1995	Uppsala	women	35-64	4,200	2,991	71.2	PQ
Men born in 1943	1993	Gothenburg	men	50	1,463	798	54.5	Q + ME
	2003	Gothenburg	men	60	749	655	87.4	Q + ME
Men and women born in 1953	2003	Gothenburg	women	50	994	668	67.2	PQ
			men		993	595	59.9	
Total					20,160	14,470	71.8	

Q + ME= questionnaires + medical examination, PQ=postal questionnaire.

Characteristics of the study population cohorts.

The Men Born in 1943 subsample consisted of a random third of 50-year-old men living in Gothenburg in 1993, re-examined in 2003, and the Women and Men Born in 1953 subsamples consisted of a random third of 50-year old women and men living in Gothenburg in 2003. The Eskil subsample consisted of a random sample of 625 men aged 30–54 and living in the city of Eskilstuna, Sweden in 1986. The Uppsala Public Health Cohort was based on random samples of 1000 women and men 25 years or older from each of the six municipalities of Uppsala County in 1993. The Beda II subsample was based on a re-examination in 1997 of a random sample drawn in 1979 of 1746 women born 1915–1941 and living in Gothenburg. The Uppsala-Örebro Women Study sample was based on random samples of 600 women aged 35–64 from each of the seven counties in the Uppsala-Örebro Health Care Region, Sweden.

All samples were by definition representative of their underlying general populations. No exclusions were made. The combined samples consisted of 20,160 subjects of whom 3,590 were part of more than one subsample. Overall, 14,470 (71.8%) observations were obtained, based on 12,000 unique individuals. Of these 10,451 (6,808 women and 3,644 men) participated once, 964 men twice, 254 men three times, and 330 men on four occasions. Additional information on the study population is given in Table 1.

### Ethical consideration

Informed consent was obtained from all participants, verbal in the early studies, and written later on, as required first by the Research Ethics Committees at Gothenburg and Uppsala Universities, and later by the National Research Ethics Board. The Committees and the Board approved the study on several occasions during the data collection process.

### Data collection

Outcome data were obtained from official registers. Other data used in this report was obtained from baseline or follow-up examinations, in some of the studies by questionnaire in connection with medical examinations performed, in others by postal questionnaires.

Educational level was classified on a four-point scale ranging from ‘compulsory education only’ (=1), to ‘college or university level education’ (=4). Employment status was measured on a four-point nominal scale as ‘gainfully employed’ (including students), ‘unemployed’, ‘on sick leave or disability pension’, or ‘old age retirement’. Marital status was classified as married/ cohabiting or not (the latter including response alternatives never married, divorced, and widowed).

Self-rated health (SRH) was measured with the Well-being subscale of The Gothenburg Quality of Life instrument (GQL) [25]. Respondents were asked to rate their health on a seven-point Likert scale with response alternatives ranging from ‘very bad’ (=1) to ‘excellent, could not be better’ (=7), and with no verbal labels of the intervening steps. The seven-point scale was used in the analyses. For illustration purposes in the Figures the seven-point scale was converted into a three point scale (1–3 (11%), 4–5 (35%), and 6–7 (54%)). Symptom reporting was assessed based on the Complaint Score subscale of the GQL, in which subjects are asked ‘Have you been troubled by any of the following symptoms during the past three months?’, followed by a list of 30 symptoms with response alternatives ‘yes’ (=1) or ‘no’ (=0) given for each symptom. The Complaint Score was obtained as the sum across the 30 symptoms.

Leisure time physical activity was reported on a four-point ordinal scale with response alternatives ‘sedentary’, ‘moderately active’, ‘active’, or ‘vigorously active’ [26]. Smoking habits were classified as ‘current smoker’ or ‘non-smoker’ (including never smoked and ex-smoker). In addition, in some of the cohort studies a five-point smoking variable was available, where smoking habits were classified as ‘never smoked’ (=1), ‘ex-smoker’ (=2), ‘currently smoking 1–14 grams of tobacco per day’ (=3), ‘smoking 15–24 grams per day’ (=4), or ‘smoking 25 grams or more per day’, one cigarette equalling 1 gram, one cheroot 2 grams, one cigar 5 grams, and pipe tobacco 50 grams divided by the number of days the pack lasted [26].

The Swedish Social Insurance Agency administers all sick leave and disability pension benefits, and their database is a complete account of official sick leave compensation and disability benefits granted. Information on all compensated days of sick leave for each individual in the study populations from 1 January 1986 until 31 December 2002 was obtained from the Agency.

The data included the first and last day of each sickness spell, the type of sick leave benefit (compensation for sickness, work injury, or rehabilitation), and extent of sick leave (25%, 50%, 75% or 100%).

Information on whether the subjects had been granted a disability pension at any time from 1971 until 2001 was obtained from the Agency. The data included decision date, diagnoses, extent (25%, 50%, 67%, 75% or 100%) and type (temporary or permanent) of disability pension. The incidence rate of new disability pension was calculated among those who did not have a disability pension at baseline.

Data on all hospital admissions from 1971 until December 31, 2002 was obtained from the National Hospital Discharge Register. The data obtained included date of admission, date of discharge and diagnoses. In this report only the main diagnosis was used. Data on cause-specific mortality from 1971 until December 31, 2002 was obtained from the National Causes of Death Register. The data used here was date of death, and underlying cause of death. The disability pension diagnoses, discharge diagnoses, and causes of death were classified according to the International Statistical Classification of Diseases and Related Health Problems (ICD) versions 8–10.

### Statistical consideration

Data was analyzed with the Statistical Analyses System software (SAS) version 9.2 [27]. Data concerning age, sex, examination year, and outcome data were complete, except for one individual where age was missing. Not all variables were measured in all subpopulations. Complaint score was not measured in the Uppsala-Örebro Women Study, and leisure time physical activity was not measured in the Eskil Study. The number of available observations for each variable is shown in Table 2. The overall proportion of missing data in subpopulations where the variables were measured was less than 2%. Missing data were not replaced. Simple differences between groups were assessed with Student’s t-test or the chi-square test.

**Table 2:** Characteristics

	**N**	**Women**			**Men**			
		**n**	**Mean or %**	**SD**	**n**	**Mean or %**	**SD**	**p**
Number of observations	14,470	6,816	47.1		7,654	52.9		
Mean follow-up time, years			8.6	2.6		11.4	5.6	
Person-years during follow up		27,034			73,217			
Age	14,469	6,816	52.3	12.6	7,653	56.5	13.0	<0.0001
Education	14,120	6,722			7,398			<0.0001
University/collage		1,605	23.9		1,554	21.0		
Upper secondary school		1,286	19.1		1,166	15.8		
Vocational school		1,608	23.9		1,905	25.8		
Compulsory school		2,223	33.1		2,773	37.5		
Employment status	14,187	6,575			7,612			<0.0001
Employed		4,444	67.6		4,511	59.3		
Unemployed		271	4.1		286	3.8		
Sick-leave/disability pension		713	10.8		624	8.2		
Old age retirement		1,147	17.4		2,191	28.8		
Married/cohabiting	14,338	4,990	74.0		5,927	78.0		<0.0001
Smoking habits	14,330	6,735			7,595			0.001
Never smoked or ex-smoker		5,002	74.3		5,455	71.8		
Current smoker		1,733	25.7		2,140	28.2		
Leisure time physical activity	13,787	6,678			7,109			<0.0001
Vigorously active		49	0.7		113	1.6		
Active		890	13.3		1,304	18.3		
Moderately active		4,663	69.8		4,511	63.4		
Sedentary		1,076	16.1		1,181	16.6		
Complaint score (range 0–30)	11,365	3,777	7.8	5.4	7,588	5.3	4.7	<0.0001
Self-rated health	14,020	6,568			7,452			<0.0001
7 “Could not be better”		1,421	21.6		2,170	29.1		
6		1,805	27.5		2,199	29.5		
5		1,486	22.6		1,373	18.4		
4		1,084	16.5		947	12.7		
3		387	5.9		423	5.7		
2		253	3.9		204	2.7		
1 “Very bad”		132	2.0		136	1.8		
Outcome during follow up								
Days of sick leave/year	6,570	3,157	18.4	78.4	3,413	17.8	79.8	0.39
Disability pension, %	6,291	507	19.6		455	12.3		<0.0001
Admitted to hospital, %	9,561	3,257	14.2		6,404	48.7		<0.0001
Deceased, %	9,561	295	9.3		1,863	29.1		<0.0001

Characteristics of the study population.

Multiple linear regression, performed with the SAS procedure ‘GLM’ providing regression coefficients, 95% confidence intervals, and Wald’s chi-square (a measure of variable impact, and p-values), was used in the analyses with the dependent variable as the outcome (number of sick leave days during follow up), and SRH and the covariates age, examination year, marital status, smoking habits, physical activity during leisure time, educational level, being unemployed, and complaint score as independent variables, with backward elimination of non-significant variables. Moreover, possible nonlinear relationships were tested with squared and cubic terms, and so were possible interaction terms but none was found.

Proportional hazards regression (Cox’s analysis) was used in the analyses of the effects of SRH at baseline on the outcome variables survival, admission to hospital, and being granted a disability pension, with the outcome entered as the dependent variable and with SRH and the same covariates as mentioned above, and being on sick leave or disability pension (regarding the outcomes hospital admission and mortality) as independent variables using the SAS procedure ‘Phreg’ providing hazards ratios (HR), 95% confidence intervals (CI), Wald’s chi-square and p-values).

The choice of independent variables potentially associated with outcome was based on variables at hand in most of the cohorts (a substantial number), and on the literature. In addition, some were chosen based on intuition. The relationship between the outcome variables and the candidate independent variables was tested in bivariate analyses stratified by sex. All variables related to an outcome in women or men were entered in the final analytical models.

Since the proportional hazards regression is dependent of proportionality between the hazard function of those exposed and those not exposed, the hazard functions across the follow-up period were calculated separately for men and women with the SAS procedure ‘Life-test’ providing tabulated and plotted hazards levels for exposed and unexposed groups. The analyses of hospital admission and disability pension were adjusted for non-exposure by censoring subjects at time of death. All tests were two-tailed. Significance levels were set at P<0.05.

## Results

### Characteristics of the study population

Study population characteristics are presented in Table 2. The study population was followed for an average of 10.5 years (range 0–24.9 years). The total number of person years during follow-up was 100,251 years, 27,034 years among women and 73,217 years among men. The mean number of days of sick leave per year among women was 18.4 and 17.8 among men, disability pension was granted to 19.6% of the women and to 12.3% of the men. Among women, 14.2%, and among men 48.7% were admitted to hospital, and 9.3% of the women and 29.1% of the men died during follow-up.

### Effects of self-rated health on outcome

**Figure 1:**
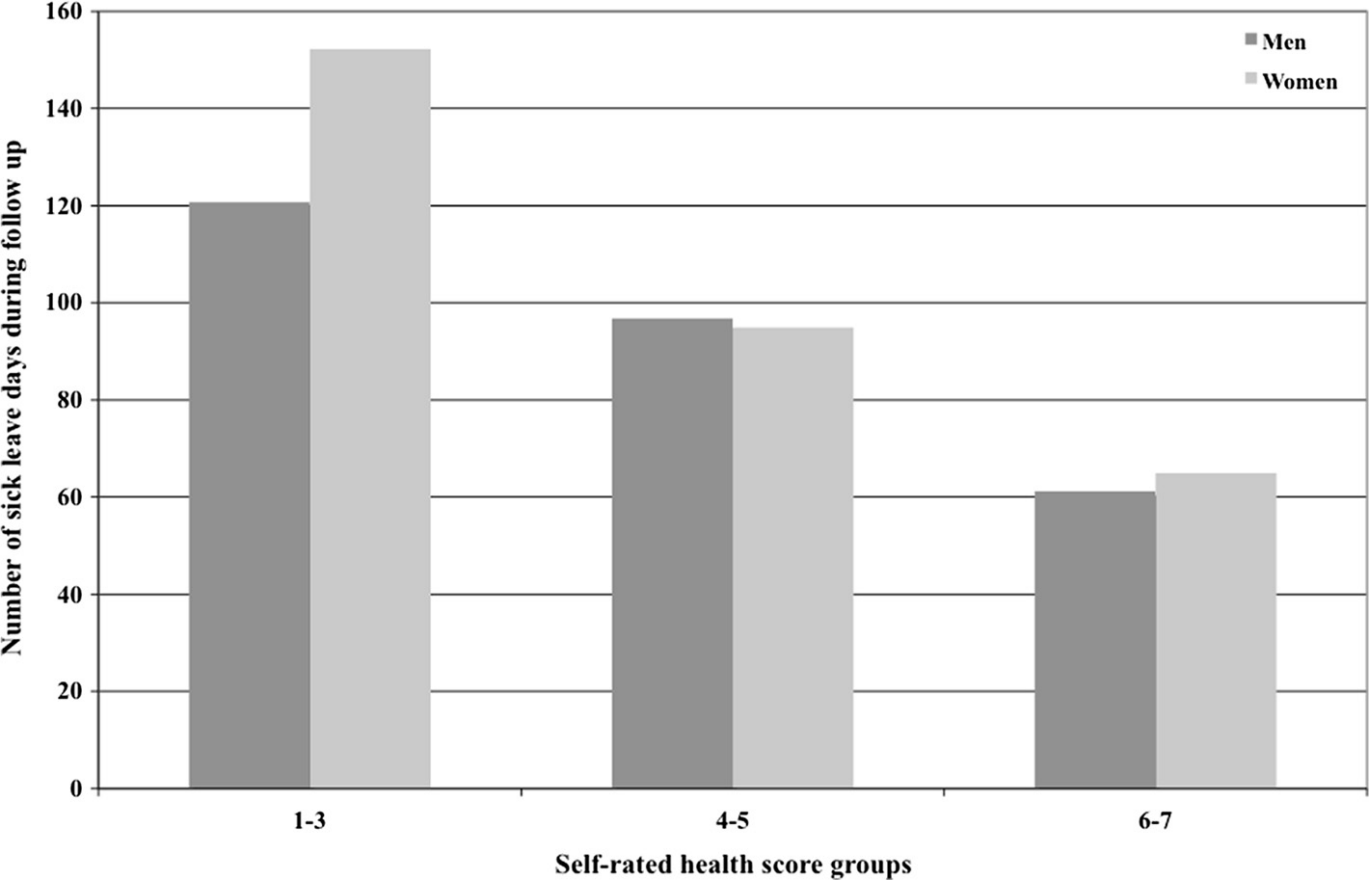
**Self**-**rated health and sick leave.** Sick leave days during follow up in groups according to self-rated health score at baseline adjusted for the influence of age, educational level, physical exercise during leisure time, smoking habits, being on sick leave or disability pension at baseline and year of examination.

Self-rated health at baseline was significantly associated with number of sick leave days during follow-up (regression coefficient −13.2, p<0.0001 among women, meaning that the number of sick leave days decreased 13.2 days by each increase in category of SRH, and −9.5, p=0.01 among men) when the effect of the covariates was taken into account (Table 3). As shown in Figure 1 women in the highest self-rated health score group had on average 65 days on sick leave during follow-up, in the middle SRH score group 95 days, and in the lowest group 152 days on sick leave during follow-up. The corresponding numbers among men were 61, 97, and 121 days.

**Table 3:** Determinants of sick leave in multivariate analysis

	**Women**			**Men**		
	**Beta**	**t**	**p**	**Beta**	**t**	**p**
SRH (1–7)	−13.2	−3.9	<0.0001	−9.5	−2.7	<0.01
Age (years)	−2.9	−9.1	<0.0001	−1.5	−1.5	<0.0001
Education (1–4)				−14.1	−3.4	<0.001
Leisure time physical activity				−17.1	−2.6	<0.01
Complaint score	4.5	4.7	<0.0001	2.4	2.3	<0.05
Year of investigation	−8.1	−3.3	<0.001	42.4	3.3	<0.001
Smoking	35.6	3.6	<0.0005	23.0	2.3	<0.05

Factors related to number of sick leave days during follow up among 2959 women and 3243 men with complete data available from 1986, based on multiple linear regression analysis.

**Figure 2:**
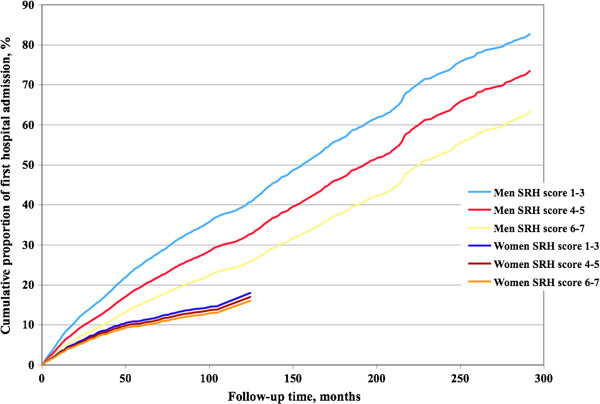
**Self**-**rated health and disability pension.** Cumulative disability pension rate among women and men in groups according to self-rated health.

Self-rated health was also significantly associated with being granted a disability pension after adjustment for the influence of the covariates. Among women the HR was 0.77 (95%CI: 0.69, 0.86, p<0.0001, Table 4, meaning that the probability of being granted a disability pension decreased by 23% by each increase in category of SRH. The corresponding number among men was 0.76 (95%CI: 0.66, 0.86, p<0.0001). Trends across follow-up time in the various self-rated health score groups are visualized in Figure 2.

**Table 4:** **Determinants of disability pension**, **hospital admission**, **and mortality**

	**Women**				**Men**			
	**HR**	**95%CI**	**χ2**	**p**	**HR**	**95%CI**	**χ2**	**p**
Disability pension								
SRH	0.77	0.69-0.86	21.0	<0.0001	0.76	0.66-0.86	17.6	<0.0001
Age	1.02	1.01-1.03	8.5	<0.005	1.04	1.03-1.06	24.3	<0.0001
Leisure-time physical activity	0.69	0.52-0.92	6.4	<0.05	0.69	0.50-0.94	5.5	<0.05
On sick-leave	7.08	4.42-11.33	66.6	<0.0001	18.4	9.9-33.9	86.4	<0.0001
Complaint score	1.08	1.05-1.12	25.5	<0.0001	1.05	1.01-1.10	5.6	<0.05
Education	ns	-	-	-	0.81	0.67-0.98	4.6	<0.05
Hospital admission								
SRH	0.96	0.90-1.02	1.6	0.20	0.87	0.85-0.89	119.5	<0.0001
Age	ns	-	-	-	1.07	1.06-1.07	1007.9	<0.0001
Year of investigation	ns	-	-	-	0.96	0.95-0.96	269.8	<0.0001
Leisure-time physical activity	0.76	0.63-0.91	8.5	<0.005	ns	-	-	-
Married	0.80	0.64-0.99	4.2	<0.05	ns	-	-	-
Smoking	-	-	-	-	1.23	1.13-1.33	24.7	<0.0001
Mortality								
SRH	0.89	0.82-0.96	8.0	<0.005	0.90	0.87-0.93	38.0	<0.0001
Age	1.12	1.10-1.13	270.8	<0.0001	1.12	1.11-1.13	878.5	<0.0001
Year of investigation	0.92	0.84-0.99	4.0	<0.05	0.99	0.98-0.99	7.0	<0.01
Leisure-time physical activity	0.59	0.45-0.78	13.8	<0.0005	0.79	0.72-0.86	28.3	<0.0001
Smoking	ns	-	-	-	1.59	1.43-1.77	75.1	<0.0001
On sick-leave /disability pension	ns	-	-	-	1.46	1.21-1.75	16.2	<0.0001

Factors related to being granted a disability pension based on 2170 women and 1646 men, being admitted to hospital based on 2944 women and 6189 men, and dying during follow up based on 2952 women 5708 men, in multivariate proportional hazards regression analysis.

**Figure 3:**
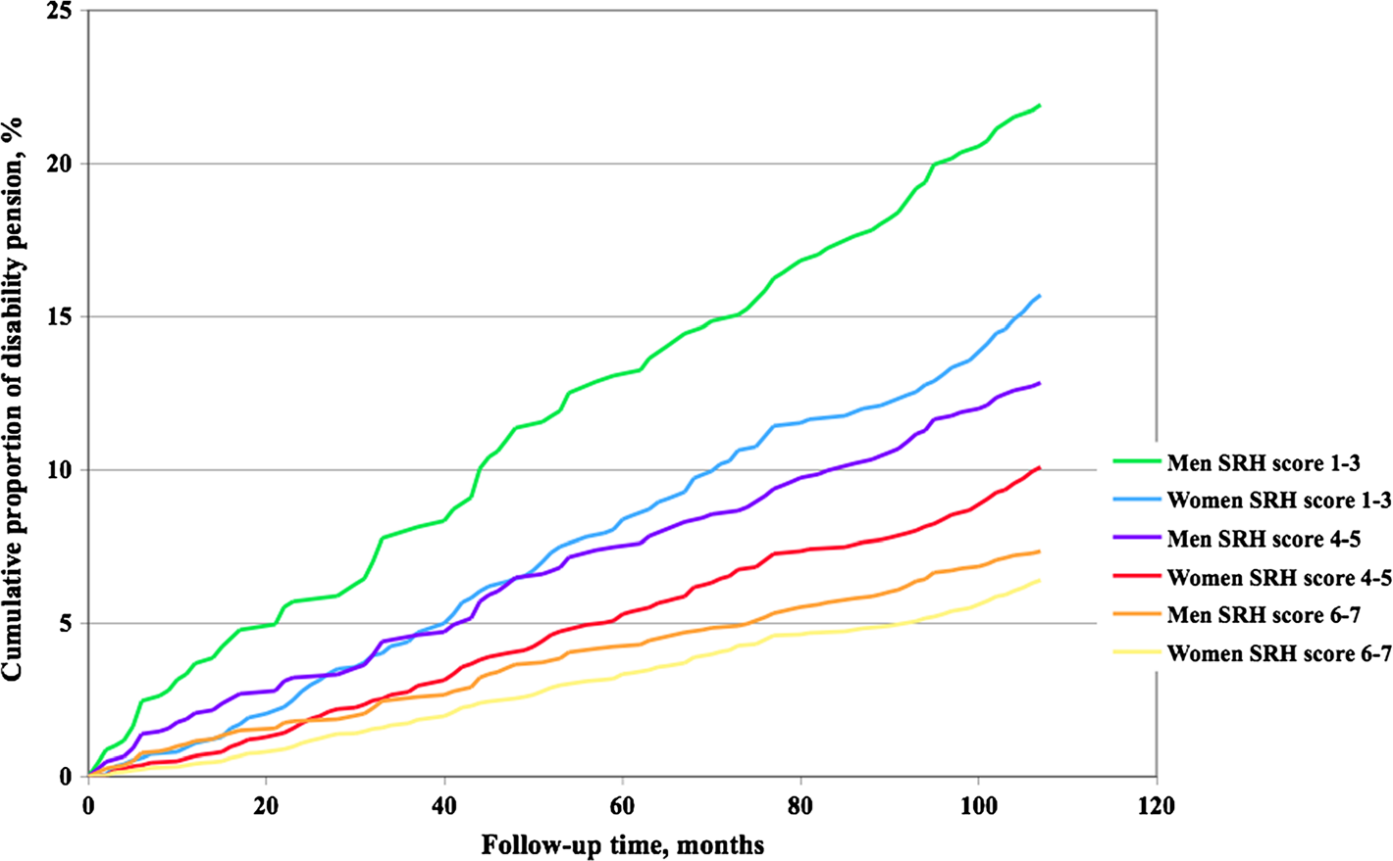
**Self**-**rated health and hospital admission.** Cumulative first hospital admission rate among women and men in groups according to self-rated health.

Among men, self-rated health was associated with hospital admission (HR 0.87, 95% CI: 0.85, 0.89, p<0.0001, Table 4, meaning that the probability of hospital admission decreased by 13% by each increase in category of SRH). Among women this association was non-significant (HR 0.96, 95% CI: 0.90, 1.02, p=0.20). The cumulative proportion of first hospital admission across follow-up time in the various self-rated health score groups are visualized in Figure 3.

**Figure 4:**
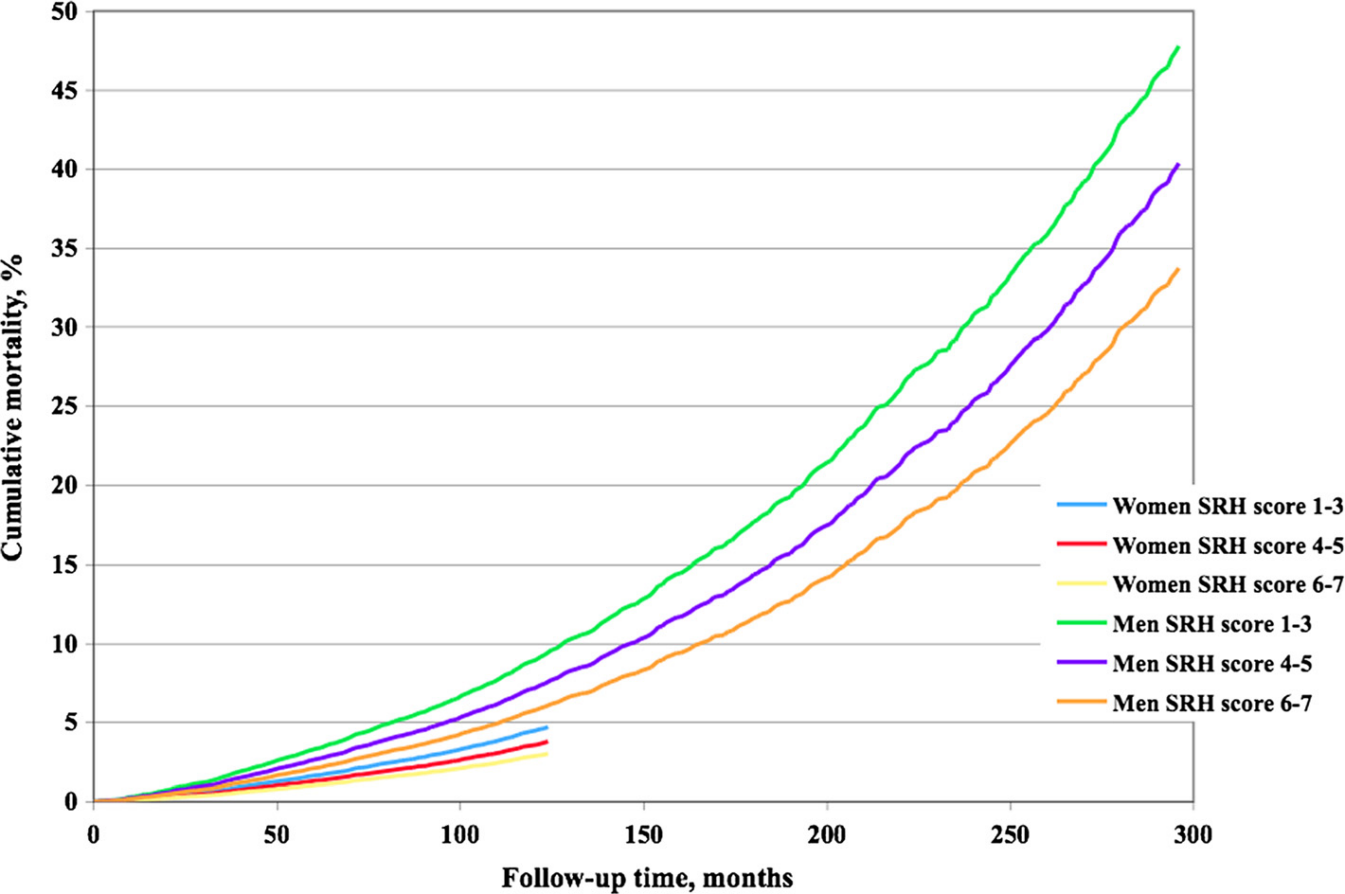
**Self**-**rated health and mortality rate.** Cumulative mortality rate among women and men in groups according to self-rated health.

Self-rated health was significantly associated with mortality during follow-up in women (HR 0.89, 95% CI: 0.82, 0.96, p<0.005, Table 4, meaning that the probability of dying decreased by 11% by each increase in category of SRH), and among men (HR 0.90, 95% CI: 0.87, 0.93, p<0.0001, Table 4). Trends across follow-up time in the various self-rated health score groups are visualized in Figure 4.

**Figure 5:**
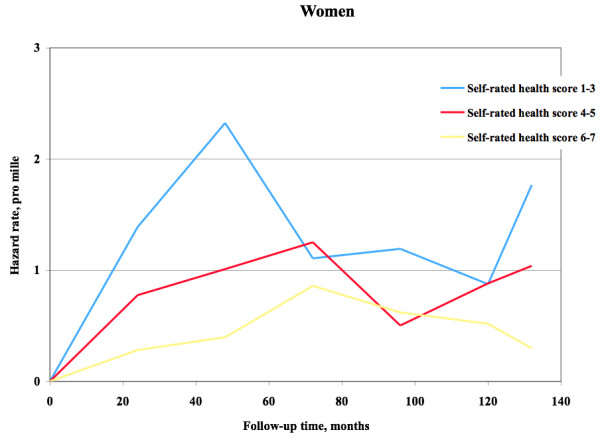
**Self**-**rated health and mortality hazard function.** Hazard function regarding mortality among women during follow up.

**Figure 6:**
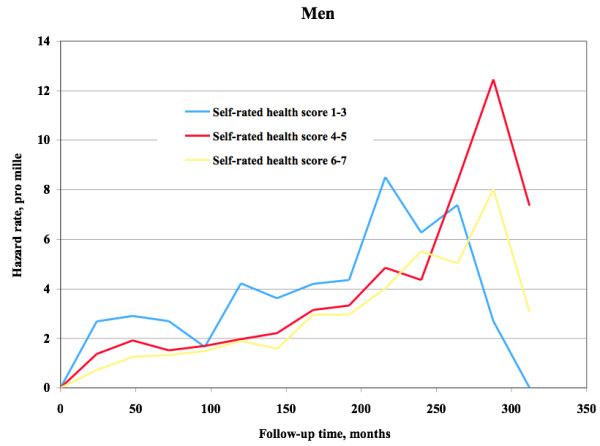
**Self**-**rated health and mortality hazard function.** Hazard function regarding mortality among men during follow up.

The hazard function for mortality among women and men is shown in Figure 5 and Figure 6, respectively. Among men there was approximate proportionality, and thereby predictive effect, during the first 225 months, corresponding to 19 years. After this point in time the remaining subjects were too few to provide stable mortality hazard levels. Among women there was proportionality during approximately 50 months, corresponding to slightly more than four years. After this point in time the remaining subjects were few.

## Discussion

In this population-based study of 11,880 women and men, there were strong inverse associations between self-rated health at baseline and sick leave, disability pension, and mortality, during follow-up. Among men, the effects were strong and consistent during follow-up, and the predictive power of SRH spanned up to three decades. Among women the effects were strong and consistent, though of shorter duration, possibly for technical reasons. Associations between SRH and hospital admission were observed in men, but not in women.

Previous research concerning associations between SRH and sick leave has mainly been based on study-populations consisting of employees from specific sectors of the work force [9,11,12] or been based on self-reported data with regards to sick-leave [10]. Present results corroborates previous findings and extends them, by using data concerning SRH from a large population based cohort of adult women and men, and official register based data with regards to sick leave. Concerning associations between SRH and disability pension, results are in line with results from previous research [13-15].

Associations between SRH and hospital admission were observed in men, but not in women. Other studies have similarly shown a higher probability for hospital admissions in men than in women [16,17]. Differences in results between women and men may in the present study be due to differences in follow-up time for women and men. Screening data concerning men were available from 1973 and onwards, while data concerning women were not available until 1993. Furthermore, there was a significant difference in age between women and men, with female participants being significantly younger than male participants, which may have affected results.

Stronger associations between SRH and short-term mortality (defined as up to two years, and up to ten years, respectively) have been reported in some studies [17,18]. These results were not corroborated in the present study where, based on analyses of HR throughout follow-up, strong associations with mortality were observed for approximately 19 years for men and for slightly more than four years for women. Whether this finding reflects a true sex difference or is an artefact attributable to exhaustion of the data set remains to be determined.

A key question in relation to public health thus concerns whether, and to what extent, trends in SRH are associated with changes in health care utilization, morbidity and mortality. Global SRH is known to encompass assessments of a wide range of health determinants [28], and may furthermore reflect norms and expectations in relation to health. A negative trend in self-rated health with gradually poorer self-ratings observed in later years of investigation was recently reported, based on data from the same study population [29]. In the present analyses, using register-based data, significant associations were observed between sick leave, disability pension, mortality, and self-rated health which remained after adjusting for covariates, including year of investigation.

Major strengths of the study are: representative large samples from the underlying general population, and use of official register data. Analyses were based on screening data from eight population-based adult cohorts and on official register data regarding sick leave, disability pension, hospital admissions, and death, with little or no data loss. The participation rate at screening was satisfactory and a survival analysis of screening participants and non-responders gave very similar results. Only validated examination instruments were used. Another strength concerns follow-up time, which for men spanned up to three decades. Moreover, analyses including the total study population, and analyses including only those who participated once, gave similar results.

Official register data covering the population as a whole were not available until 1986, for data concerning sick leave and disability pension. Follow-up time available for analyses concerning sick leave and disability pension was therefore shorter, than follow-up time concerning hospital admissions and mortality.

The main limitation of the study concerns the difference between women and men with regards to age and in terms of when screening data were collected. Male cohorts were screened from 1973 and onwards, while female cohorts were screened from 1993 and onwards. Results concerning differences in the association between SRH and hospital admission, or mortality, between women and men should be interpreted with caution.

## Conclusions

Self-rated health has strong predictive validity in relation to use of social insurance facilities and health care services, and to mortality. Associations are strong and robust, and in men may last for up to three decades.

## Competing interests

The authors declare that they have no competing interests.

## Authors’ contributions

TW, AR, AB, SJ, HE and KS performed the data collection in their respective datasets. CH and KS performed the analyses. CH and KS drafted the manuscript. All authors participated in the critical discussions of the results, identification of relevant reference literature, and in the revisions of the manuscript. All authors read and approved the final manuscript.

## Pre-publication history

The pre-publication history for this paper can be accessed here:

http://www.biomedcentral.com/1471-2458/12/1103/prepub
